# To reconnect or not reconnect distal Purkinje fibers, that is the question when modeling the Purkinje fiber network

**DOI:** 10.3389/fphys.2025.1657611

**Published:** 2025-09-01

**Authors:** Jason D. Bayer, Karli Gillette, Ruben Coronel, Gernot Plank, Edward J. Vigmond

**Affiliations:** ^1^ Electrophysiology and Heart Modeling Institute, IHU Liryc, Fondation Bordeaux Université, Pessac-Bordeaux, France; ^2^ Institut de Mathématiques de Bordeaux, UMR5251, University of Bordeaux, Bordeaux, France; ^3^ Scientific Computing and Imaging Institute, University of Utah, Salt Lake City, UT, United States; ^4^ Department of Biomedical Engineering, University of Utah, Salt Lake City, UT, United States; ^5^ Department of Biophysics, Medical University of Graz, Graz, Austria; ^6^ Department of Experimental Cardiology, Amsterdam University Medical Centers, Location AMC, Amsterdam, Netherlands

**Keywords:** Purkinje network, conduction system, modeling, simulation, retrograde activation, anterograde activation, arrhythmia

## Abstract

**Background and aims:**

Multiple rule-based approaches exist to model the structure of the His-Purkinje system (HPS). While some approaches reconnect Purkinje fibers in the Purkinje fiber network, others do not. The aim of this study was to determine the impact of distal Purkinje fiber reconnections on anterograde activation, retrograde activation, and reentrant arrhythmias.

**Methods:**

In a human biventricular model with or without distal Purkinje fiber reconnections, normal sinus rhythm was simulated by His bundle pacing (anterograde activation), followed by an S1S2 protocol applied to the right ventricular apex (retrograde activation). Activation times in the myocardium and HPS were compared for both anterograde and retrograde HPS activation. Arrhythmia vulnerability windows and duration were determined by identifying the S1S2 coupling intervals that induced a reentry of at least two full rotations. Arrhythmia maintenance was further studied by inducing reentry with 4 Hz line pacing applied to the left ventricular epicardial surface. Reentry duration for each protocol was determined over a 20 s window. The S1S2 and line pacing protocols were repeated in the biventricular model without an HPS.

**Results:**

Anterograde activation times and arrhythmia initiation vulnerability windows were mostly unaltered when removing distal Purkinje fiber reconnections. However, retrograde activation times were 18% longer in the HPS and 8% longer in the myocardium when removing distal Purkinje fiber reconnections. Reentrant arrhythmias from the S1S2 protocol and rapid line pacing lasted longer for the model with (11.2 and >20 s) versus without (3.2 and 8.2 s) distal Purkinje fiber reconnections. The S1S2 protocol did not induce reentrant arrhythmias in the human ventricles model without an HPS, and reentry induced with 4 Hz line pacing lasted only 3.6 s.

**Conclusion:**

Retrograde activation times increased and the duration of reentrant arrhythmias shortened in the absence of Purkinje fiber reconnections in the Purkinje fiber network. This could be an important structural HPS property to incorporate into computational heart models when investigating retrograde activation and/or reentrant arrhythmias. Modifying the structure of the Purkinje fiber network to remove Purkinje fiber reconnections in patients with life threatening ventricular arrhythmia might be antiarrhythmic.

## Introduction

The His-Purkinje system (HPS) is the ventricular component of the cardiac conduction system. It is composed of the His bundle with major fascicles that bifurcate into left and right bundle branches. At the distal regions of each bundle branch is the Purkinje fiber network, which is a complex structure of Purkinje fibers that electrically couples to the myocardium through Purkinje-Muscular Junctions (PMJs).

In the original description of the Purkinje fiber network by Tawara et al. (Tawara, 1906), it was shown to have Purkinje fibers that branch (bifurcate) and reconnect (converge) within the network. This was later confirmed by India ink injection, transparent specimens, and computed tomography (De Almeida et al., 2015). During the developmental stages of the heart, the expression of Nkx2-5 in the myocardium promotes this meshing of the Purkinje fibers into the Purkinje fiber network (Park and Fishman, 2017).

In computer simulations of the cardiac conduction system (Stephenson et al., 2017), it may be important to accurately model this mesh structure of the Purkinje fiber network within the HPS. In particular, it may play a critical role in the generation of activation patterns during normal sinus rhythm, ventricular pacing, and/or reentrant arrhythmias (Durrer et al., 1970). Unfortunately, it is difficult to model the complex structure of the HPS in its entirety in three dimensions from imaging alone (Peirlinck et al., 2021). Consequently, it is commonly reconstructed in human ventricular models using rule-based approaches (Liu and Cherry, 2015; Vigmond and Clements, 2007; Ijiri et al., 2008; Al-Nashash and Lvov, 1997; Atkinson et al., 2011).

Rule-based approaches for generating the HPS structure can differ noticeably in the Purkinje fiber network. Specifically, some rule-based approaches develop a mesh structure for the Purkinje fiber network by reconnecting distal Purkinje fibers (Gillette et al., 2021; Behradfar et al., 2014), while others have a tree structure without reconnecting distal Purkinje fibers (Costabal et al., 2016; Álvarez-Barrientos et al., 2025). In other words, the latter approach develops an HPS with unique pathways from the His bundle to the PMJs with only branching of Purkinje fibers. The impact of this difference on anterograde activation, retrograde activation, and reentrant arrhythmia initiation/maintenance is unknown. We hypothesize that modeling the Purkinje fiber network with reconnecting Purkinje fibers facilitates simulating activation times and reentrant behavior observed in patients.

The main objective of this study was to determine the impact of distal Purkinje fiber reconnections on anterograde activation, retrograde activation, and reentrant arrhythmia initiation/maintenance. To accomplish this, we utilized an established computer model of the human ventricles including an HPS (Bayer et al., 2022), and performed simulations in this model with the same major fascicles and PMJs either with or without reconnections of Purkinje fibers in the Purkinje fiber network. This computational study demonstrates that distal Purkinje connections may play an important role in retrograde activation and arrhythmia maintenance, but not anterograde activation.

## Methods

### Human biventricular model

The electrical activation of ventricular myocardium coupled to the HPS was investigated using an established computer model of the human ventricular conduction system (Bayer et al., 2022). The full details on the geometry and electrophysiology of the non-failing human ventricles can be found in (Bayer et al., 2016). The full details on the structure and electrophysiology of the HPS can be found in Behradfar et al. (2014), Bayer et al. (2022). In short, the parameters governing cellular and tissue electrophysiology in this anatomically accurate human model of the ventricular conduction system were fit to experimental and clinical data in order to reproduce physiologic depolarization and repolarization patterns that generate the human ECG (Durrer et al., 1970). Specifically, the human biventricular model includes transmural and apicobasal heterogeneity in cellular coupling, calcium handling, and ionic channel currents that generate the physiological depolarization and repolarization patterns intrinsic to human ventricular myocardium. These properties were essential to include since they can impact electrical conduction and arrhythmogenesis in the human heart (Han et al., 2021).

To introduce an arrhythmic substrate into this otherwise healthy heart model, the maximal conductance of the slow delayed rectifier potassium current (G_Ks_) in the HPS was decreased from the value of 0.98 pS/pF as in Walton et al. (2014) to its default ventricular myocyte value of 0.392 pS/pF (Tusscher et al., 2004). The G_Ks_ parameter was chosen since it directly influences APD in the model and has been linked to ventricular arrhythmias (Varshneya et al., 2018). Importantly, the APD generated from this modification to G_Ks_ (maximum of 371 ms) was within the physiological data range reported for Purkinje fibers of non-diseased human hearts (Nagy et al., 2015). With this modification, at normal sinus rhythm rates action potential duration in the HPS is longer than in the myocardium on average by 56 ms across all PMJs in the model. This promotes unidirectional conduction block at short stimulus coupling intervals during the S1S2 programmed stimulation protocol described below. To prevent arrhythmia dynamics from being dependent on variations in PMJ density (Behradfar et al., 2014), the PMJ density of the HPS in the myocardium was fixed to 15 PMJs per cm^3^ with a junctional resistance of 100 kΩ.

### HPS structure with or without distal Purkinje fiber reconnections

**FIGURE 1:**
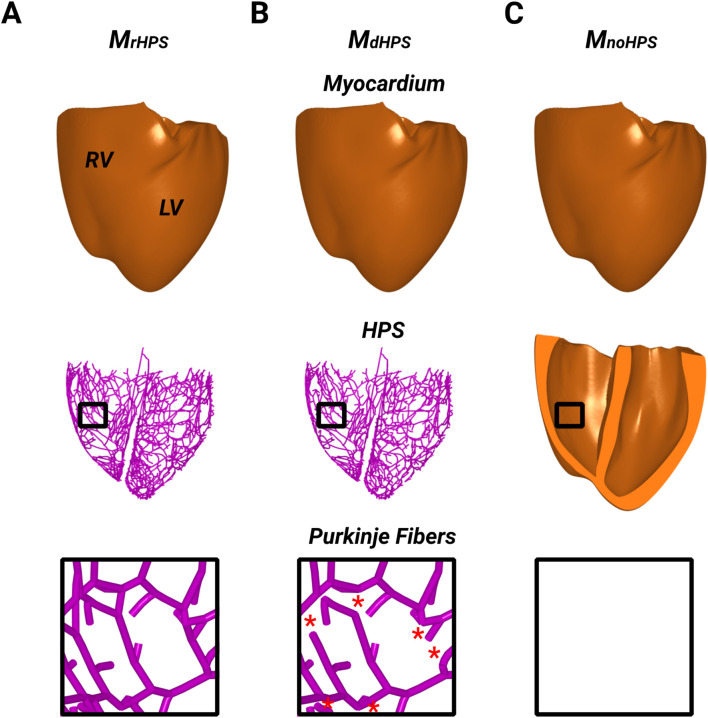
The three model cases showing the ventricular myocardium with reconnected distal Purkinje fibers **(A)**, disconnected distal Purkinje fibers **(B)**, and without the HPS **(C)**. Black boxes were placed in the RV of each model to zoom in on the Purkinje fiber structure to show the presence or absence of distal Purkinje fiber reconnections. Red stars in the inset of **(B)** indicates where distal Purkinje fiber reconnections were disconnected (removed) from the inset in **(A)**.

The structure of the HPS in the biventricular model was modified to exclude reconnections between Purkinje fibers within the Purkinje network of the left and right bundle branches (Figure 1). Specially, cross-bridging fibers between the major ascending fibers were disconnected to convert the meshed HPS structure to a tree structure. At each reunification point with two parent nodes, i.e., where two parent cables connected to one child, the second parent cable was removed up to the point where it originated at a bifurcation. Thus, no PMJs were left unconnected to a parent branch after these branch removals, while the major fascicles and bundle branches remained intact. As a result, there were two test cases to study anterograde and retrograde activation. The first test case was the human ventricles model with an HPS that had reconnected Purkinje fibers within the Purkinje fiber network (M_rHPS_, Figure 1A), and the second was the human ventricles with the same HPS but with disconnected Purkinje fibers within the Purkinje fiber network (M_dHPS_, Figure 1B). A third test case was used to investigate how the HPS contributes to reentrant arrhythmia initiation and/or maintenance. This case was the same human ventricles model as used for the other two cases, but with the HPS removed (M_noHPS_, Figure 1C).

### Anterograde activation

The His bundle was paced at a cycle length (CL) of 750 ms for 10 cycles. Activation times (ATs) were computed at each node of the myocardial and HPS meshes for the last cycle of pacing as the moment when the action potential upstroke exceeded the voltage threshold of −10 mV. Differences in the activation patterns between the two test cases in Figure 1 were quantified using the method of Han et al. (Han et al., 2012). Using this approach, the relative difference (RD), root mean square difference (RMSD) and correlation coefficient (CC) were computed for the comparisons M_dHPS_ versus M_rHPS_ and M_noHPS_ versus M_rHPS_.

### Retrograde activation

Following His pacing, the right ventricular endocardial apex was paced (S1) at a CL of 600 ms for 10 cycles from a spherical region with a diameter of 1.5 mm. This stimulus diameter was chosen to mimic a standard 5F catheter size used for clinical pacing studies. Activation times (ATs) were computed and compared in the same manner as previously described for anterograde conduction. Note, the CL of 600 ms was chosen since it is a commonly used CL for programmed electrical stimulation in cardiac electrophysiology laboratories. This CL is also within the range used to investigate retrograde activation (Sung et al., 1981). Since this CL mimics a heart rate slightly higher than normal resting heart rates (100 bpm vs. 60–90 bpm), it prevents sinus rhythm activation originating from the atria from interfering with S1 capture. Since APD restitution of the ventricular myocardium in the model does not steepen until S1 CL < 500 ms (120 bpm) (Bayer et al., 2016), retrograde activation patterns were not dependent on our choice of S1 CL > 500 ms.

### Arrhythmia initiation

Following S1 pacing, premature S2 stimuli were applied at the same RV pacing site with a CI beginning at 400 ms, which mimics programmed electrical stimulation used to induce ventricular arrhythmias in cardiac electrophysiology laboratories. The S1S2 CI was then gradually reduced by decrements of 10 ms until a loss of stimulus capture. All pacing stimuli were administered with a 2 ms duration at a strength of twice the diastolic threshold. The diastolic stimulus threshold was determined for each new set of model parameters. Following each S2 stimulus, the transmembrane voltage maps of the ventricular myocardium and HPS were inspected for unidirectional conduction block and reentry that lasted for more than 2 full rotations. In other words, reentry was identified when a point in the HPS or ventricles was activated more than once following the S2. The vulnerable window of reentry was recorded by identifying the first and last S2 coupling intervals that generated reentry.

### Arrhythmia maintenance

In addition to studying arrhythmia maintenance following reentries from the S1S2 protocol, reentry was induced with rapid line pacing using the protocol from our previous computational and animal studies (Moreno et al., 2022; Moreno et al., 2019; Bayer et al., 2024). This protocol consistently induces the same reentry in each of the three models to unbiasedly study how the arrhythmic substrate maintains the induced arrhythmia. In short, pacing was administered from an apicobasal line electrode 2 mm in diameter on the left ventricular epicardium by stimulating the entire line at 8x the diastolic stimulation threshold with a pacing CL of 400 ms for 10 cycles. The 8x capture threshold strength ensured homogeneous activation across the entire line. Subsequently, reentry was induced with rapid pacing from the same line electrode with a pacing CL of 250 ms for 10 cycles at 8x the diastolic stimulation threshold. Reentry was verified by visual inspection of the transmembrane voltage maps in the HPS and/or myocardium. The duration of this reentry, as well as the reentry induced for the longest S2 coupling interval from the S1S2 protocol, was recorded over a 20 s window until the reentry self-terminated or the end of the arrhythmia observation window was reached.

### Simulation platform

Monodomain simulations were performed using the cardiac electrophysiology simulator CARP-EP (numericor.at) running in parallel on 512 cores. For this study, simulations for the preconditioning normal sinus rhythm protocol, S1S2 protocol, and rapid line pacing protocol were performed for the 2 cases with different HPS structures and the case without an HPS. These simulations had a computational cost of 34,560 CPU hours on 512 cores of the high-performance analytics and computing platform IRENE (Joliot-Curie) at the TGCC supercomputing center. All simulations used a time step of 20 μs, and their results were visualized using the software Meshalyzer (https://git.opencarp.org/openCARP/meshalyzer). Stimuli for all protocols were administered by transmembrane current injection with diastolic stimulation thresholds obtained by increasing the stimulus strength in increments of 1 μA/cm (starting from zero) until action potential propagation was initiated in the myocardium from the stimulus site.

## Results

### Anterograde activation

**FIGURE 2:**
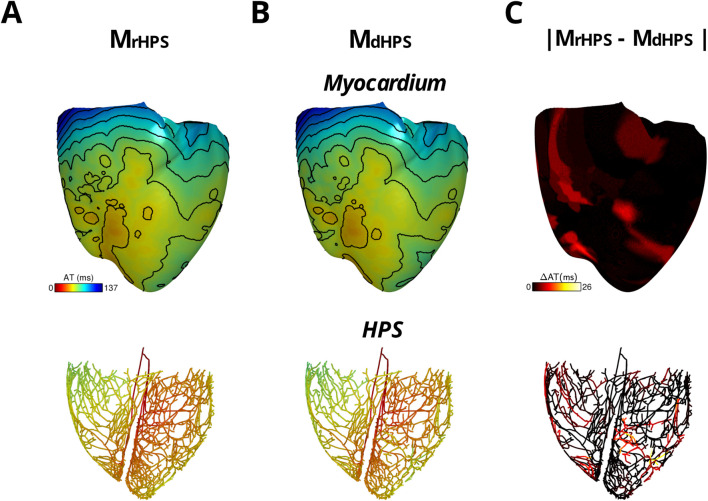
The two model cases with an HPN showing anterograde activation in transmembrane voltage maps of the HPS and myocardium either with reconnected distal Purkinje fibers **(A)** or disconnected distal Purkinje fibers **(B)** Isoline spacing is 10 ms. Activation times ranged from 21 to 136 ms in the myocardium and 0–67 ms in the HPS of **(A)** and 21–137 ms in the myocardium and 0–70 ms in the HPS of **(B)** The absolute difference in activation times between the two cases is shown in **(C)**.

For each test case in Figure 1, activation times were computed at the end of the normal sinus rhythm protocol to identify differences in anterograde conduction in the ventricular myocardium and HPS. Figure 2 shows the total activation times in the HPS and the myocardium for the models with distal Purkinje fiber reconnections (M_rHPS_, Figure 2A) and without distal Purkinje fiber reconnections (M_dHPS_, Figure 2B). Minor differences were observed in the isolines between the activation maps in the ventricular myocardium between cases M_rHPS_ and M_dHPS_ (compare Figure 2A with Figure 2C). To quantify these differences, the RD, RMSD, and CC were computed for the comparison between the M_dHPS_ and M_rHPS_ models (Table 1).

**TABLE 1:** Comparison of anterograde and retrograde activation times between the models with reconnected distal Purkinje fibers and disconnected distal Purkinje fibers.

	%|∆ in total AT|	RD (%)	RMSD (ms)	CC
*Anterograde* *M*_*dHPS*_ *vs. M*_*rHPS*_				
Myocardium	0.87	0.03	1.36 ± 1.43	0.99
HPS	4.41	0.08	3.21 ± 3.26	0.97
*Retrograde* *M*_*dHPS*_ *vs. M*_*rHPS*_				
Myocardium	7.86	0.09	7.24 ± 5.08	0.98
HPS	18.18	0.20	8.95 ± 6.12	0.95

For the comparison of the M_dHPS_ and M_rHPS_ models, in the myocardium for the HPS with disconnected distal Purkinje fibers, total activation time increased by only 1 ms, while the absolute change in total AT was less than 1% with the RMSD only slightly larger than a millisecond. In the HPS, these values were larger by only a few milliseconds. The CC values ≥ 0.97 showed a very strong linear relationship between the activation patterns for these two test cases. Figure 2C shows the distribution in the absolute difference in activation times between the two models.

The impact of using different G_Ks_ values between the default (0.98 pS/pF) and arrhythmogenic (0.392 pS/pF) values on anterograde activation was unnoticeable. For any value within this range differing by 0.1 pS/pF, we did not find changes >1 ms to anterograde activation times nor activation patterns. This was due to the CV restitution curves of the HPS and myocardium being flat at the His pacing CL of 750 ms.

### Retrograde activation

**FIGURE 3:**
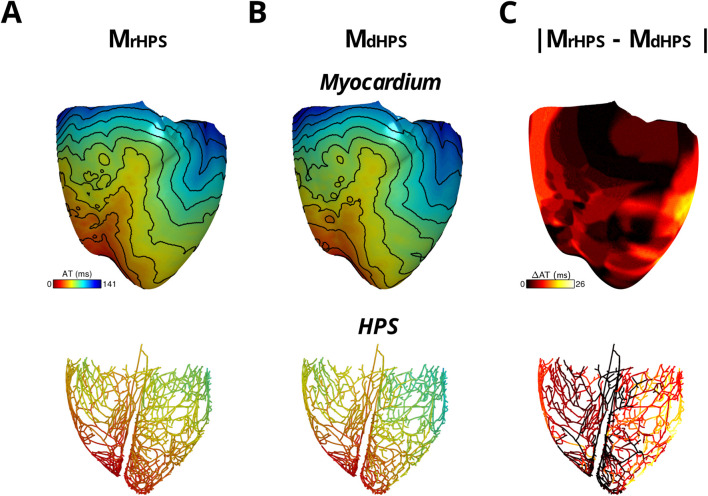
The two model cases with an HPN showing retrograde activation in transmembrane voltage maps of the HPS and myocardium either with reconnected distal Purkinje fibers **(A)** or disconnected distal Purkinje fibers **(B)**. Isoline spacing is 10 ms. Activation times ranged from 0 to 130 ms in the myocardium and 8–80 ms in the HPS of **(A)** and 0–141 ms in the myocardium and 8–96 ms in the HPS of **(B)**. The absolute difference in activation times between the two cases is shown in **(C)**.

For each test case in Figure 1, activation times were computed at the end of S1 pacing to identify differences in the retrograde activation of the ventricular myocardium and HPS. Figure 3 shows the total activation times in the HPS and the myocardium for the models M_rHPS_ Figure 3A and M_dHPS_ (Figure 3B). In contrast to anterograde conduction, differences in the activation maps between Figures 3A,B appeared larger in the ventricular myocardium and HPS. To quantify these differences, the RD, RMSD, and CC were computed in the same manner as for anterograde activation in order to compare between the M_dHPS_ and M_rHPS_ models (Table 1).

For the comparison of the M_dHPS_ and M_rHPS_ models, in the myocardium of M_dHPS_ total activation time increased by 11 ms, while the absolute change in total AT was 7.86% with an RMSD of 7.25 ms. In the HPS of M_dHPS_ compared to M_rHPS_, the increase in total activation time was larger by 16 ms along with a larger absolute change in total AT of 18.18% and an RMSD of 8.95 ms. The CC values ≥ 0.95 still showed a strong linear relationship between the activation patterns for these two test cases. Figure 3C shows the distribution in the absolute difference in activation times between the two models.

The impact of using different G_Ks_ values between the default (0.98 pS/pF) and arrhythmogenic (0.392 pS/pF) values on retrograde activation was also unnoticeable. For any value within this range differing by 0.1 pS/pF, we did not find changes >1 ms to retrograde activation times nor activation patterns. This was due to the CV restitution curves of the HPS and myocardium being flat at the His pacing CL of 600 ms.

### Arrhythmia initiation

**FIGURE 4:**
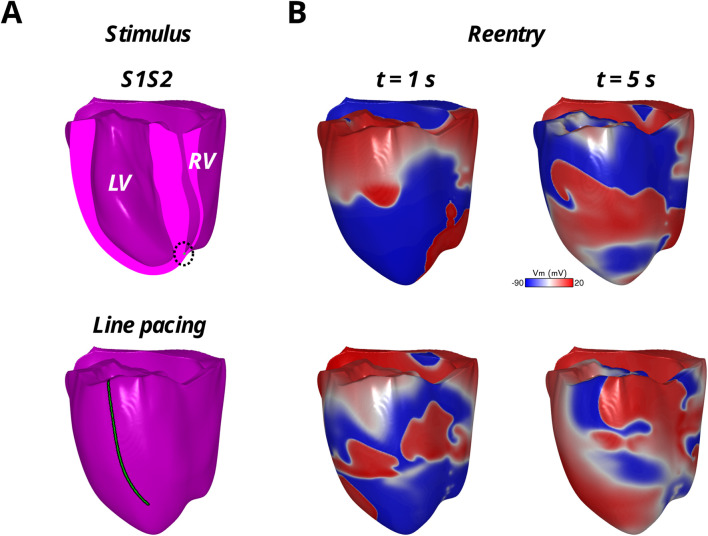
Reentrant arrhythmias in the model with reconnected distal Purkinje fibers with the S1S2 protocol applied to the endocardial RV apex (top row) and the rapid LV epicardial line pacing protocol (bottom row). The stimulus locations are shown in green in **(A)** and the reentrant arrhythmia in the transmembrane voltage maps (Vm) are shown in **(B)** at two time points during the first 5 s of reentry.

Results for the arrhythmias induced with the S1S2 protocol applied to the RV endocardial apex of the three test models are shown in Table 2. Reentry was initiated in M_rHPS_ and M_dHPS_, but not M_noHPS_. The vulnerable windows for the test cases M_rHPS_ and M_dHPS_ were similar (S1S2 coupling intervals from 270 to 320 ms). Within the arrhythmia vulnerability windows for M_rHPS_ and M_dHPS_, reentry was induced by unidirectional conduction block that lasted for at least 2 full rotations (Figure 4).

**TABLE 2:** Arrhythmia initiation and maintenance of reentry following the S1S2 and rapid line pacing protocols.

	M_rHPS_	M_dHPS_	M_noHPS_
*Initiation*			
Last S2 capture	260	260	260
First reentry after S2 (ms)	320	320	—
Last reentry after S2 (ms)	270	270	—
*Maintenance*			
Reentry duration following S2 = 320 ms (s)	11.2	3.2	—
Reentry duration following line pacing (s)	>20	8.2	3.6

### Arrhythmia maintenance

Arrhythmia maintenance was quantified as the duration of the reentry that was visible in the myocardium and/or HPS after the administration of rapid line pacing, as well as for reentry following the S1S2 coupling interval of 320 ms. The duration of these arrhythmias can be found in Table 2. Note, rapid line pacing initiated reentrant arrhythmias in all three models, with an example shown for M_rHPS_ in Figure 4. The reentries in all three models started out as a figure-of-eight reentry from conduction block along the electrode placed along the apicobasal axis of the ventricles. After a few seconds the behavior of the reentry changed with respect to the structure or presence of the HPN. Specifically, these reentries lasted for the entire 20 s observation window in M_rHPS_ and less than 9 s in M_dHPS_. Reentry was even shorter in M_noHPS_ lasting less than 4 s. Similar results were observed for the S1S2 coupling interval of 320 ms, where reentry duration was 3.2 s for M_dHPS_ and the entire 20 s observation period for M_rHPS_.

## Discussion

This study used a state-of-the-art computational model of the human ventricles and HPS to identify how Purkinje fiber reconnections in the Purkinje fiber network influence anterograde activation, retrograde activation, and reentrant arrhythmias. This *in silico* study demonstrates that retrograde activation patterns and arrhythmia maintenance can be impacted by distal Purkinje fiber reconnections, while antegrade activation patterns and arrhythmia initiation are not. In the model with disconnected distal Purkinje fibers, the total retrograde activation time increased by 8% in the myocardium and 18% in the HPS compared to the model with reconnected distal Purkinje fibers. Reentrant arrhythmias from the S1S2 protocol and rapid line pacing lasted longer in the model with reconnected (11.2 and >20 s) versus disconnected (3.2 and 8.2 s) distal Purkinje fibers.

### The dependence of anterograde conduction on Purkinje fiber network structure was weak in the model

Numerous studies have implemented the rule-based approaches mentioned in the introduction (Liu and Cherry, 2015; Vigmond and Clements, 2007; Ijiri et al., 2008) into digital twinning pipelines (Gillette et al., 2021; Behradfar et al., 2014; Costabal et al., 2016). These pipelines aim to reproduce patient-specific activation patterns and ECGs during normal sinus rhythm. Regardless of the choice of the rule-based algorithm to generate HPS structure, they have shown success for reproducing physiological activation patterns underlying the ECG during normal sinus rhythm.

The universal success of these approaches indicates that reproducing the detailed structure of the Purkinje network may not be critical for generating patient activation patterns and QRS durations during normal sinus rhythm. In other words, changing the structure of the Purkinje network should not significantly impact activation patterns on the endocardium of the ventricles as long as the macrostructure of the HPS is modeled appropriately.

Figure 2 and Table 1 of our study show that when removing reconnections in the Purkinje fiber network, the difference in total anterograde activation time without Purkinje fiber network reconnections was only a few milliseconds. Therefore, we concluded that the generation of the initial endocardial activations observed in the human ventricles by Durrer et al. (1970) is more dependent on the major fascicles of the left and right bundle branches in the model than on the topology of the Purkinje fiber network. Thus, efforts should be focused more on the macrostructure of the left and right bundle branches in the HPS if the only goal is to simulate anterograde activation patterns in the ventricles. Furthermore, this independence on the algorithm used to generate HPS structures for normal sinus rhythm provides users with the flexibility to choose from a wider array of rule-base approaches that best fits their needs. For example, methods that simplify the HPS into a thin fast-conducting endocardial layer are computationally inexpensive and have shown promise for simulating sinus rhythm activation (Okada et al., 2018). Such approaches might be more practical to employ for studies that require a large number of sinus rhythm simulations to be performed in a large number of heart models.

### The dependence of retrograde activation on Purkinje fiber network structure was stronger in the model

Few studies have investigated how Purkinje fiber network structure impacts retrograde activation patterns and reentrant arrhythmias in intact human ventricles (Bayer et al., 2022; Bayer et al., 2024). Our *in silico* study demonstrates that retrograde activation from ventricular pacing, ectopic foci, or reentrant arrhythmias can differ from anterograde activation depending on the structure of the Purkinje fiber network. This is due to electrical activation from the myocardium being able to re-enter the HPS at multiple locations at different times.

Our study suggests that reconnected Purkinje fibers in the Purkinje fiber network can be an essential feature to model when investigating retrograde activation in ventricles during ventricular pacing and reentrant arrhythmias. In models with disconnected distal Purkinje fibers, we expect that corroborating simulation results with animal and/or clinical data will be more challenging. Thus, drawing conclusions on activation patterns simulated during ventricular pacing and reentrant behaviors observed in models without distal Purkinje fiber reconnections should be done with extreme caution.

Imaging data supports the need for modeling a Purkinje fiber network with Purkinje fiber reconnections (Stephenson et al., 2017; Ono et al., 2009; Cha et al., 2020). From these studies, it is clear that distal Purkinje fibers reconnect. Furthermore, the Purkinje network is generated from a contiguous endocardial layer of specialized myocardium during the early stages of development (Sedmera and Gourdie, 2014). Therefore, the branching and reconnection of distal Purkinje fibers is to be expected. Due to the impact of the Purkinje fiber network structure on retrograde activation maps and arrhythmia behavior, future studies should focus on modeling more accurately both the macrostructure and microstructure of the HPS, as well as making rule-based HPS algorithms more flexible to include new rules for HPS structure as imaging studies evolve.

### Arrhythmia initiation and maintenance

Our *in silico* study suggests that arrhythmia initiation is less dependent on the structure of the Purkinje network than arrhythmia maintenance. This makes sense since the initiation of arrhythmias using the protocols from this study relies heavily on the presence of repolarization heterogeneity for induction. Specifically, the S1S2 protocol relies on the action potential duration heterogeneity across the PMJs to generate unidirectional conduction block and reentry (Behradfar et al., 2014; Martinez et al., 2018; Haissaguerre et al., 2016). For the rapid line pacing protocol, it relies on the apicobasal action potential duration heterogeneity in the ventricular myocardium to generate unidirectional conduction block and reentry (Bayer et al., 2016). Therefore, it is not surprising that distal Purkinje fiber reconnections had little effect on arrhythmia initiation, i.e., the first few rotations of a reentry generated from unidirectional conduction block.

As the reentrant arrhythmia persisted, the role of both anterograde and retrograde conduction within the HPS became important regardless of the arrhythmia initiation protocol that was used (Figure 4; Table 2). A previous study by our group showed that defibrillation success using low-energy approaches relies heavily on eliminating reentrant pathways through the HPS (Bayer et al., 2022). We also showed that rapidly pacing the His bundle helps to eliminate HPS reentrant pathways, where at specific frequencies this caused the reentrant arrhythmia to terminate (Bayer et al., 2024). Therefore, when there are more Purkinje fiber reconnections within the Purkinje fiber network, the number of possible reentrant pathways in the HPS should also increase to promote the maintenance of reentrant arrhythmias. Future work will investigate the mechanism of specific types and locations of distal Purkinje reconnections regarding these pathways in the HPN.

Further supporting the role of the Purkinje network in lethal ventricular arrhythmias, it has been shown that removing Purkinje activation sites with radiofrequency ablation is anti-arrhythmic (Coronel et al., 2021). Thus, modifying the structure of the Purkinje fiber network to remove Purkinje fiber reconnections in patients with life threatening ventricular arrhythmia may be antiarrhythmic. This could theoretically be done with gene therapy (Wang et al., 2025), ablation (Imnadze and Zerm, 2019), or pacing (Bayer et al., 2024). Based on our recent findings and this body of work on the role of the Purkinje network in ventricular arrhythmias, further imaging studies should investigate how Purkinje fiber reconnections within the Purkinje fiber network vary between patients, and if patients with more distal Purkinje fiber reconnections are more susceptible to arrhythmias.

The propensity of sustained ventricular arrhythmias in the model with the mesh HPS structure may be explained by the microstructure of the HPS. For example, there could be current source-sink mismatches during retrograde activation of the HPS when thinner Purkinje fibers merge into a single thicker Purkinje fiber. When these current source-sink mismatches become large enough, which would be expected at fast rates of activation, they could lead to unidirectional conduction block and sustained reentry if the surrounding HPS architecture and coupled myocardium provide a suitable pathway to support reentry (Ciaccio et al., 2018). Since the model with the mesh HPS structure has more interconnections between Purkinje fibers than the tree HPS structure, this may explain its higher propensity towards sustained ventricular reentrant arrhythmias. Future studies are warranted to identify the occurrence and conditions for these current source-sink mismatches to occur in the Purkinje network of the HPS, in addition to determining the specific micro-architectures in the finer branches of the HPS that are able to support sustained reentry. To validate this study, imaging data will be required to verify the existence of these micro-architectural structures.

In addition to HPS microstructure, there is evidence that spatial heterogeneity exists in the repolarization of the HPS which could impact HPS-mediated reentrant arrhythmias. In isolated Purkinje/myocardial tissue preparations from canine and human ventricles, APD prolongs from the His bundle to the distal regions of the Purkinje network, and then shortens again closer to PMJs (Myerburg et al., 1970). In optical mapping studies using *ex vivo* rabbit hearts (Logantha et al., 2021), APD is longer near the His bundle than in distal Purkinje fibers. Consequently, this spatial heterogeneity in APD could impact the initiation and/or maintenance of the reentries observed in our human ventricles model with the HPS. To address this issue, future studies are planned with our model to investigate the mechanisms of HPS-mediated reentrant arrhythmias in relation to spatial heterogeneities in the HPS from calcium handling, ion channel currents, and cellular coupling.

### Clinical significance

The findings of this study may have clinical significance to noninvasive therapies for ventricular tachyarrhythmias, such as Sterotactic body radiotherapy (SBRT) (Blanck et al., 2020). When anti-arrhythmic drugs and radiofrequency ablation fail to terminate and prevent the reoccurrence of ventricular tachyarrhythmias, SBRT is an alternative for permanently removing the arrhythmic substrate. During SBRT the entire myocardium is exposed to doses of radiation in the range of 15–40 Gy, which in turn has been shown in rats to increase conduction velocity, shorten action potential duration, and increase the peak of the calcium transient in the ventricular myocardium (Mages et al., 2025). These changes are antiarrhythmic in cardiac tissue by reducing the wavelength of reentry (APD*CV). Furthermore, SBRT is non-selective and likely reduces the APD gradient across the PMJs, which our studies suggest could be antiarrhythmic. Future simulation studies will hopefully provide valuable mechanistic insight into the effectiveness of SBRT for treating lethal ventricular arrhythmias.

### Limitations

Due to the computational expense of performing simulations in the human model of the ventricular conduction system, we only used a single human ventricular geometry and HPS to investigate anterograde activation, retrograde activation, and reentrant arrhythmias. To speed up simulation times and reduce computational costs, using eikonal approaches as done for solving anterograde ventricular activation maps (Gillette et al., 2021) is not practical for retrograde activation maps and reentry in the HPS. We plan to expand this study to multiple hearts, both healthy and diseased, when new approaches arise that are capable of achieving this feature. Specifically, we till target hearts with fibrosis, scars, and age/sex-related changes in cardiac electrophysiology and geometry. Secondly, we artificially introduced the arrhythmic substrate for S1S2 programmed stimulation to induce reentrant arrhythmias by decreasing G_Ks_ in the HPS. For now, we deem this acceptable since little is known regarding the structure and function of the HPS in diseased hearts. When such data becomes available in the future, this study will be expanded to include specific pathological abnormalities in the structure and electrophysiology of the HPS. We will also thoroughly perform a sensitivity analysis of our results to a wider range of GKs values than used for this study. Thirdly, we used a fixed 15 PMJs per cm^3^ density in the human ventricles model. According to the study by Behradfar et al. (Behradfar et al., 2014), increasing PMJ density above this value is not expected to impact arrhythmia dynamics, but reducing it below 13 PMJs per cm^3^ could. Thus, future studies are warranted to investigate the impact of lower PMJs densities in the model on the arrhythmia outcome of our study. Fourth, we only performed programmed electrical stimulation using a clinical S1S2 protocol administered to the right ventricular endocardial apex for studying retrograde activation and arrhythmogenesis. It is possible that retrograde activation and arrhythmia initiation/maintenance could be impacted by the pacing location in the ventricles. In future studies we plan to investigate how various cardiac resynchronization therapy setups impact retrograde activation patterns and arrhythmogenesis. Fifth, we assume that the HPS structure at the left and right ventricular apex is continuous with the lower portion of the septum. We also assume that only major fascicles without PMJs exist in the middle to upper sections of the right ventricular septum. This is notably different from other approaches constructing rule-based HPSs for digital twinning purposes (Gillette et al., 2021). The impact of these differences in HPS structure on retrograde activation and arrhythmogenesis will also be explored in future studies and validated with imaging and electrophysiology data from human hearts when available. Lastly, our simulation study lacked mechano-electric and neural feedback. While it is acknowledged that both can influence activation patterns and arrhythmogenesis in the human ventricles, they were not included in our study at this time in order to keep the computational expenses and complexity of our simulations manageable. When possible and appropriate to do so, they will be included in future studies to help refine the model and provide confidence to the simulation results.

## Conclusion

Retrograde activation times increased and the duration of reentrant arrhythmias shortened in the absence of distal Purkinje fiber reconnections observed in histological and imaging studies. The reconnection of distal Purkinje fibers could be an important structural HPS property to incorporate into computational heart models when investigating retrograde activation and/or reentrant arrhythmias. Modifying the structure of the Purkinje fiber network to remove Purkinje fiber reconnections in patients with life threatening ventricular arrhythmia may be antiarrhythmic.

## Data Availability

The raw data supporting the conclusions of this article will be made available by the authors, without undue reservation.
